# HADLN: Hybrid Attention-Based Deep Learning Network for Automated Arrhythmia Classification

**DOI:** 10.3389/fphys.2021.683025

**Published:** 2021-07-05

**Authors:** Mingfeng Jiang, Jiayan Gu, Yang Li, Bo Wei, Jucheng Zhang, Zhikang Wang, Ling Xia

**Affiliations:** ^1^School of Information Science and Technology, Zhejiang Sci-Tech University, Hangzhou, China; ^2^Department of Clinical Engineering, The Second Affiliated Hospital, School of Medicine, Zhejiang University, Hangzhou, China; ^3^Department of Biomedical Engineering, Zhejiang University, Hangzhou, China

**Keywords:** arrhythmia classification, deep learning, bidirectional LSTM, ResNet, attention mechanism

## Abstract

In recent years, with the development of artificial intelligence, deep learning model has achieved initial success in ECG data analysis, especially the detection of atrial fibrillation. In order to solve the problems of ignoring the correlation between contexts and gradient dispersion in traditional deep convolution neural network model, the hybrid attention-based deep learning network (HADLN) method is proposed to implement arrhythmia classification. The HADLN can make full use of the advantages of residual network (ResNet) and bidirectional long–short-term memory (Bi-LSTM) architecture to obtain fusion features containing local and global information and improve the interpretability of the model through the attention mechanism. The method is trained and verified by using the PhysioNet 2017 challenge dataset. Without loss of generality, the ECG signal is classified into four categories, including atrial fibrillation, noise, other, and normal signals. By combining the fusion features and the attention mechanism, the learned model has a great improvement in classification performance and certain interpretability. The experimental results show that the proposed HADLN method can achieve precision of 0.866, recall of 0.859, accuracy of 0.867, and F1-score of 0.880 on 10-fold cross-validation.

## Introduction

Atrial fibrillation is one of the most common persistent arrhythmias. It is characterized by irregular atrial activity, increasing incidence rate, and associated complications, such as stroke and systemic thromboembolism, which pose a great threat to human health and life (Mathew et al., 2009). In addition, due to the lack of comprehensive understanding of the pathological mechanism of atrial fibrillation, the timely diagnosis of atrial fibrillation becomes a problem (Wyndham, 2000). People often miss the optimal treatment time because the early stages of atrial fibrillation are usually paroxysmal and asymptomatic (Mehall et al., 2007). Therefore, the development of a new type of automatic atrial fibrillation detection system to provide accurate and reliable diagnostic information as early as possible is of great significance for improving the quality of treatment and reducing the further deterioration of the patient’s health.

Electrocardiography (ECG) is often used for routine monitoring of physiological signals in clinical application. The effective analysis of ECG signals is helpful to detect many heart diseases such as atrial fibrillation (AF), myocardial infarction (MI), and heart failure (HF) (Turakhia, 2018). In an AF waveform, the P wave is replaced by many inconsistent fibrillatory waves, and the RR interval is irregular, which is easily mixed with other diseases (Wei et al., 2017). In the early stage, the research work of ECG classification was generally implemented by using manual feature extraction method. However, the method of manual feature extraction was not only affected by noises but also lost a lot of important information, which cause the in accuracy and low efficiency of AF classification. Moreover, its poor generalization ability cannot be used to deal with the practical application. Some signal processing methods, such as independent component analysis (Prasad et al., 2013), discrete wavelet transform (Lee et al., 2013), and entropy (Liu et al., 2018a), has been used to improve the performances of manual feature extraction. Recently, feature extraction methods based on machine learning, such as support vector machine (Liu et al., 2018b) and random forest (Kennedy et al., 2016), are proposed to classify the ECG signals.

Recently, deep neural networks (DNNs) achieved initial success in ECG data processing (Parvaneh et al., 2019), which can provide another opportunity to improve the accuracy and scalability of automatic ECG classification obviously (Hong et al., 2019). According to different network structure, DNNs can integrate different level features and classifiers to form an end-to-end multilayer model (Dang et al., 2019) without preprocessing a large amount of data by manual rules, which can overcome the limitation of traditional machine learning algorithm model with independent input and output (Schmidhuber, 2015). In addition, there have been some new attempts on DNNs, such as residual blocks (He et al., 2016), deep convolutional neural network (Wu et al., 2020), deep residual convolutional neural network (Li et al., 2020), recurrent neural network (RNN) with long–short-term memory (LSTM) (Faust et al., 2018), and deep bidirectional LSTM (Bi-LSTM) network (Yildirim, 2018). In order to effectively select feature information and enhance the interpretability of the model, the attention mechanisms had been valued in the classification of arrhythmia (Yao et al., 2020; Zhang et al., 2020). In the PhysioNet/Computing in Cardiology Challenge 2020, several classification models related to attention mechanisms have been proposed to get promising classification results. Duan et al. (2020) proposed a multiscale attention deep neural network (MADNN) method to boost capability of extracting the ECG features on different scales, combining kernel- and branch-wise attention modules, which can achieve an overall score of 0.446 on the hidden testing-set. Liu et al. (2020) proposed a novel multilabel classifier of 12-lead ECG recordings by using residual CNN and class-wise attention mechanism, which can get resulting scores of 0.5501 ± 0.0223 according to the challenge metric, demonstrating a promising method for the classification of ECGs. He et al. (2020) used the mechanism of attention to learn an attention distribution on the list of extracted features, and then, the attention weightings were integrated into a single feature vector and used for the final classification. The overall score with five cross-validation of training set is 0.543 by using the Deep Heart model, demonstrating that it may have potential practical applications. However, there still a long way to improve classification accuracy in clinical application.

This paper proposed a hybrid attention-based deep learning network (HADLN) method to automatically implement ECG classification. The PhysioNet 2017 challenge data were used to validate the performance of HADLN method. The main contributions of this paper can be concluded as follows: (1) the ResNet part uses the superposition of 16 residual blocks to extract local features, and the bidirectional long-short-term memory network was used to extract the global features in parallel. Moreover, the global feature from Bi-LSTM and the local feature from ResNet were the fused features, which can extract multiple features of the original ECG data; (2) in this paper, a modification of the standard attention mechanism was proposed to strengthen local feature information from ResNet according to the weight parameters calculated from fused features; and (3) the features of these weighting parameters based on fused features can proved a interpretability for ECG classification results.

## Basic Theory

In this paper, three deep-learning approaches are utilized to form the classification model. Residual network (ResNet) and Bi-LSTM network are applied in the classification model. Besides, attention mechanism is introduced to improve the performance of classification.

### Bi-LSTM

**FIGURE 1:**
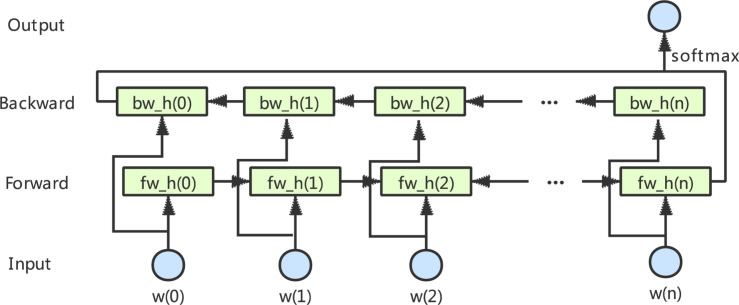
The architecture of bidirectional LSTM.

LSTM is a typical RNN proposed by Hochreiter and Schmidhuber (1997). Due to the advantages of its gate mechanism, it is easier to learn the long-term dependencies between sequences (Tan et al., 2018). The bidirectional layer is actually composed of two LSTM layers in opposite directions: the forward LSTM layer and the backward LSTM layer. The Bi-LSTM architecture is shown in Figure 1, which will be able to fully consider the global features in the input data. Graves and Schmidhuber showed that such bidirectional networks can be significantly more effective than unidirectional LSTM architectures (Graves and Schmidhuber, 2005).

### ResNet

**FIGURE 2:**
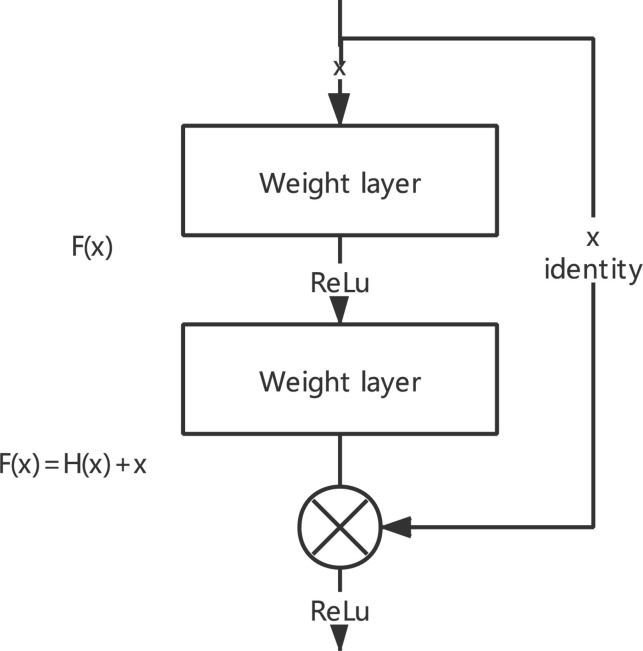
Principle of the residual module.

The deep CNN network with residual blocks can solve the problem of the convergence difficulty of the deep network and overcome the problem of network degradation caused by the increase in network layers (Zagoruyko and Komodakis, 2016). As shown in Figure 2, the learning process is to let multiple nonlinear computing layers of continuous stack fit the residual F(x) = H(x) − X between the input data and the output data. Residual learning adds a shortcut on the basis of the traditional linear network structure, which is integrating a shortcut with the main path by the method of additive fusion.

### Attention Mechanism

**FIGURE 3:**
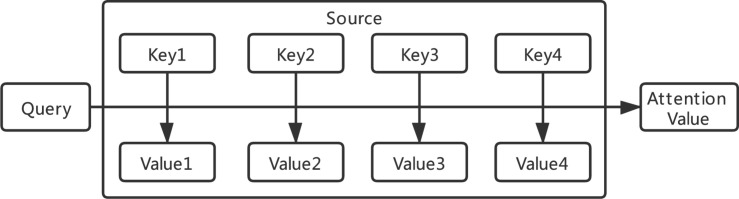
Attention principle architecture.

The core concept of attention mechanism is to simulate human attention mechanism to improve the performance of deep learning (Mnih et al., 2014). By using the probability distribution of attention, we can control the weighting parameters of the elements in the input sequence to generate the output sequence. As shown in Figure 3, the essence of the attention function can be described as a mapping from a query to a series of key-value pairs. The common similarity functions are implemented by multiplication in Equation 1, concatenation in Equation 2, and perceptron in Equation 3.

(1) $$f\left(Q,K_{i}\right)=Q^{T}W_{a}K_{i}$$

(2) $$f\left(Q,K_{i}\right)=W_{a}\left[Q:K_{i}\right]$$

(3) $$f\left(Q,K_{i}\right)=v_{a}^{T}tanh\left(W_{a}Q+U_{a}K_{i}\right)$$

where *W*_*a*_, *U*_*a*_, and *v*_*a*_ are all learnable parameters. *Q* means Query, and *K*_*i*_ means keys.

## Materials and Methods

### Dataset

To demonstrate the generalizability of the proposed HADLN architecture, the open dataset of the PhysioNet 2017 challenge was applied in the model (Clifford et al., 2017), which contained four rhythm categories: normal (N), atrial fibrillation (A), other (O), and noise (∼). The dataset consisted of 8,528 single lead ECG data recordings, and each of them is sampled at 300 Hz with a length of 9–61 s. The dataset was divided into a training set (90%) and a testing set (10%) for training and evaluation in all tasks. Data profile of PhysioNet Challenge 2017 dataset is shown in Table 1.

**TABLE 1:** Data profile of PhysioNet challenge 2017 dataset.

Type	# recording	Time length (s)				
		Mean	StDev	Max	Median	Min
Normal	5,154	31.9	10.0	61.0	30	9.0
AF	771	31.6	12.5	60	30	10.0
Other rhythm	2,557	34.1	11.8	60.9	30	9.1
Noisy	46	27.1	9.0	60	30	10.2
Total	8,528	32.5	10.9	61.0	30	9.0

### Proposed HADLN Architecture

**FIGURE 4:**
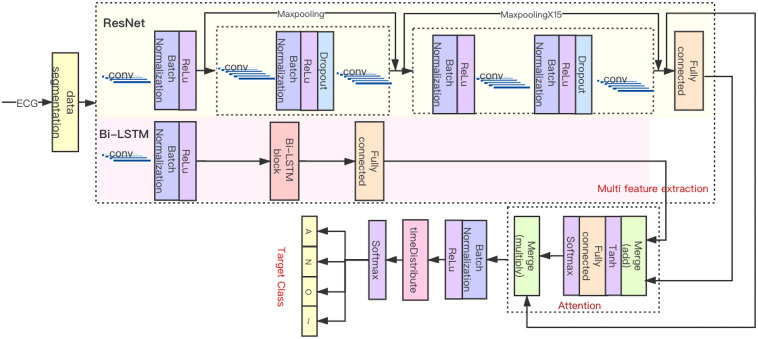
HADLN architecture.

As shown in Figure 4, the HADLN architecture was proposed to automatically detect atrial fibrillation based on the fusion of attention mechanism and deep learning model, which combines ResNet, Bi-LSTM, and attention mechanism module. The ResNet part uses the superposition of 16 residual blocks to extract local features, which can effectively solve the problem of gradient dispersion while increasing the number of network layers. At the same time, the bidirectional long–short-term memory network was used to extract the global features in parallel, and the number of units in the layer is set to 128. The global feature from Bi-LSTM and the local feature from ResNet are used to fuse the hybrid feature. Then, the weighting parameter in attention mechanism is calculated according to hybrid features by using Softmax. Finally, the weighted features are proposed to implement ECG classification.

The original ECG signal is input into several initial layers, and the output feature map is subsequently processed by 16 residual blocks sequentially including 33 convolution layers and 16 maximum pool layers. There are two types of residual modules, including two 1D convolutional layers, batch normalization layer, ReLU activation layer, dropout layer, and a maxpooling layer. As shown in Table 2, each convolutional layer has 32 × 2^k^ convolution kernels (where k starts out as 0 and is incremented every fourth). The difference is that the 2nd to 16th residual blocks have more batch normalization layers, ReLU activation layer, and dropout layers than the first residual block. The residual module combines the output of the quick connection and the output of the second convolutional layer by summation. When the feature map passes through the maxpooling layer with a pool size of 2, the length of that will be halved. When the pool size is 1, there is no effect on the feature map, so only eight layers play a role in this part of ResNet. Therefore, the original input is finally subsampled by a factor of 2^8^, and after the local feature extraction part, the output length is 1/256 of the input length.

**TABLE 2:** The length/number of convolution kernels and pool size of max-pooling layers in each residual module.

ResNet module	Kernel length	Kernel number	Pool size
1	16	32	1
2	16	32	2
3	16	32	1
4	16	32	2
5	16	64	1
6	16	64	2
7	16	64	1
8	16	64	2
9	16	128	1
10	16	128	2
11	16	128	1
12	16	128	2
13	16	256	1
14	16	256	2
15	16	256	1
16	16	256	2

For long sequences, Bi-LSTM can be used to process input along the time sequence in a parameters-sharing manner and utilizes their internal state to memorize the context. The original signal is input to Bi-LSTM to extract global features, where the number of LSTM units in each of the forward and backward layers was set to 128. The global feature h_*i*_ from Bi-LSTM and the local feature v_*i*_ from ResNet are used to fuse the hybrid feature *e*_*i*_, as shown in Equation 4. The weighting parameter *α_*i*_* in attention mechanism is calculated by using Equation 5, and the weighted features *S*_*HADLN*_ are proposed to implement ECG classification; specific implementation is shown in Equation 6.

(4) $$e_{i}=W_{a}^{T}*\text{tanh}\left(W_{Q}*v_{i}+W_{k}*h_{i}\right)$$

(5) $$\alpha_{i}=softmax\left(e_{i}\right)=\frac{\text{exp}\left(e_{i}\right)}{\sum_{i=1}^{T}\text{exp}\left(e_{i}\right)}$$

(6) $$S_{HADLN}=\sum_{i=1}^{T}\alpha_{i}*v_{i}$$

where *e_i_* the is merged feature from *h_i_* and *v_i_*, with fully connected layer parameters *W_Q_*, *W_k_*, $W_{a}^{T}$, and α_*i*_ referring to weight parameters from Softmax function, and *S*_*HADLN*_ refers to weighted features.

The classification part consists of batch normalization layer, timeDistributed layer, and two activation layers. The ReLU layer enables the classification part to accelerate the back propagation of gradients. The timeDistributed layer is fully connected in the time dimension. The second activation layer is a Softmax layer, which outputs the predicted probability distribution of four classes, including atrial fibrillation, noise, other, and normal.

As a comparison, the ResNet model with attention mechanism, termed as ResNet_A method, is proposed for ECG classification. The output of ResNet *v*_*i*_ is directly used to calculate the weighting parameters *α_*i*_*′ by Softmax function in Equation 7, and then the weighting parameters are used to calculate the weighted features in Equation 8.

(7) $$\alpha_{i}^{\prime }=softmax\left(v_{i}\right)=\frac{\text{exp}\left(v_{i}\right)}{\sum_{i=1}^{T}\text{exp}\left(v_{i}\right)}$$

(8) $$S_{ResNet\_A}=\sum_{i=1}^{T}\alpha_{i}^{\prime }*v_{i}$$

### Model Training

Batch normalization is used to ensure the smooth convergence of the network before each convolution layer. Meanwhile, using the ReLU activation function can effectively improve the learning efficiency of the network and significantly reduce the number of iterations required for convergence in the deep learning network. The initial learning rate of the Adam optimizer was set to 10^–2^ and the probability of dropout is set as 0.3. The cross-entropy function was used to evaluate the difference between the output and reference labels, as in Equation 9. The smaller the value of cross-entropy is, the closer the distribution of actual output and expected output is. According to the cross entropy, the stop mechanism in the model training can be made. When the cross-entropy value does not change in eight epochs, then the model training will stop automatically.

(9) $$loss\left(X,r\right)=-\log\frac{\text{exp}\left(P\left(X,r\right)\right)}{\sum_{i=0}^{N}\text{exp}\left(P\left(X,i\right)\right)}$$

where *r* refers to label, and *P*(*X*,*i*) is the probability the model assigns the label *i* to the input *X*.

Moreover, the HADLN and several comparative experiments were trained and tested in a server with Tesla v100-sxm2 GPU. The deep learning model was programmed by using Python 3.6 and Keras 2.1.6 framework. Matplotlib tools are used for data visualization, and numpy1.18.1 is used for a large number of dimensional arrays and matrix operations. In addition, we used scikit-learn 0.22.1 for data mining and data analysis tools.

## Results

### Performance Metric

In order to evaluate the performance of the proposed model, the precision, recall, and accuracy are listed as the following equations, respectively. The counting rules for the numbers of the variables are listed as shown in Table 3. In addition, the performance metric F1-score proposed by 2017 Physionet challenge was used to evaluate the performance of the proposed HADLN network architecture, as shown in the Equation 17.

(10) $$precision=\frac{TP}{TP+FP}$$

(11) $$recall=\frac{TP}{TP+FN}$$

(12) $$accuracy=\frac{TP+TN}{TP+TN+FP+FN}$$

(13) $$F_{1n}=\frac{2N_{n}}{\left(\Sigma \text{n}+\Sigma N\right)}$$

(14) $$F_{1a}=\frac{2A_{a}}{\left(\Sigma \text{a}+\Sigma A\right)}$$

(15) $$F_{1o}=\frac{2O_{o}}{\left(\Sigma \text{o}+\Sigma O\right)}$$

(16) $$F_{1p}=\frac{2P_{p}}{\left(\Sigma \text{p}+\Sigma P\right)}$$

(17) $$\mathrm{F1}\text{-}\mathrm{score}=\frac{\left(F_{1n}+F_{1a}+F_{1o}+F_{1p}\right)}{4}$$

**TABLE 3:** Counting rules for the numbers of the variables.

	Normal	AF	Other	Noisy	Total
Normal	Nn	Na	No	Np	Σ*N*
AF	An	Aa	Ao	Ap	ΣA
Other	On	Oa	Oo	Op	ΣO
Noisy	Pn	Pa	Po	Pp	ΣP
Total	Σn	Σa	Σo	Σp	

where TP means true positive, the number of AF signals classified correctly; FP means false positive, the number of AF signals classified wrongly; TN means true negative, the number of signals without AF classified correctly; and FN means false negative, the number of signals without AF classified wrongly.

### Experimental Results

**FIGURE 5:**
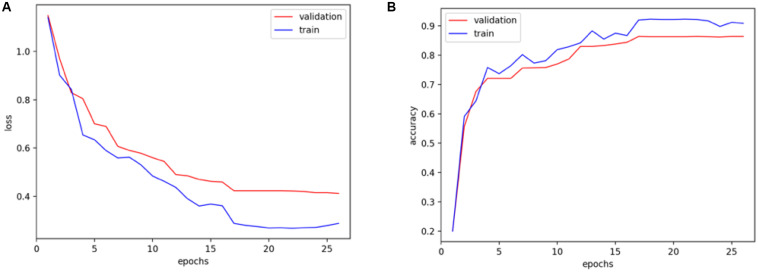
Training and validation of **(A)** loss function and **(B)** accuracy over the epochs.

As shown in Figure 5, the performance of the training set is slightly better than that of the validation set, and the model converges to a stable value, indicating that the parameters are not excessive when training the model. In the validation model, the proposed method works well, which can achieve the stable classification results with good accuracy.

In order to validate the performances of the proposed HADLN method, several state-of-the-art methods, such as ResNet (Hannun et al., 2019), CL3 (Warrick and Homsi, 2017), QRS-LSTM (Maknickas, 2017), and Dense-net (Rubin et al., 2017), are also provided as a comparison. In addition, self-attention based ResNet method, ResNet_A, is also investigated for arrhythmia classification. As shown in Table 4, the precision, recall, F1-score, and accuracy of different DNNs architecture are presented for classifying normal (N), atrial fibrillation (A), other (O), and noise (∼). It can be found that the proposed HADLN method can achieve the best classification performances with the highest metric indexes among these methods. In addition, in order to validate the robustness of the proposed HADLN method, the classification performances (F1 score, precision, recall, accuracy) have been reported in the Table 5, which indicates that the proposed HADLN method has stable classification in different cross cases.

**TABLE 4:** Classification results of weight average.

	F1-score	Precision	Recall	Accuracy
CL3	0.856	0.856	0.850	0.867
QRS-LSTM	0.666	0.770	0.714	0.770
Dense-net	0.843	0.867	0.860	0.860
ResNet	0.837	0.865	0.853	0.857
ResNet_A	0.844	0.854	0.853	0.853
HADLN	0.880	0.866	0.859	0.867

**TABLE 5:** The classification performances of the proposed HADLN method using 10-fold cross.

No.	F1-score	Precision	Recall	Accuracy
1	0.857	0.862	0.857	0.865
2	0.850	0.865	0.856	0.860
3	0.880	0.873	0.872	0.872
4	0.887	0.890	0.879	0.890
5	0.905	0.884	0.885	0.891
6	0.887	0.877	0.876	0.888
7	0.879	0.840	0.827	0.836
8	0.911	0.839	0.833	0.837
9	0.900	0.870	0.859	0.861
10	0.848	0.858	0.850	0.867
Average	0.880	0.866	0.859	0.867
Standard deviation	0.021	0.016	0.018	0.019

**FIGURE 6:**
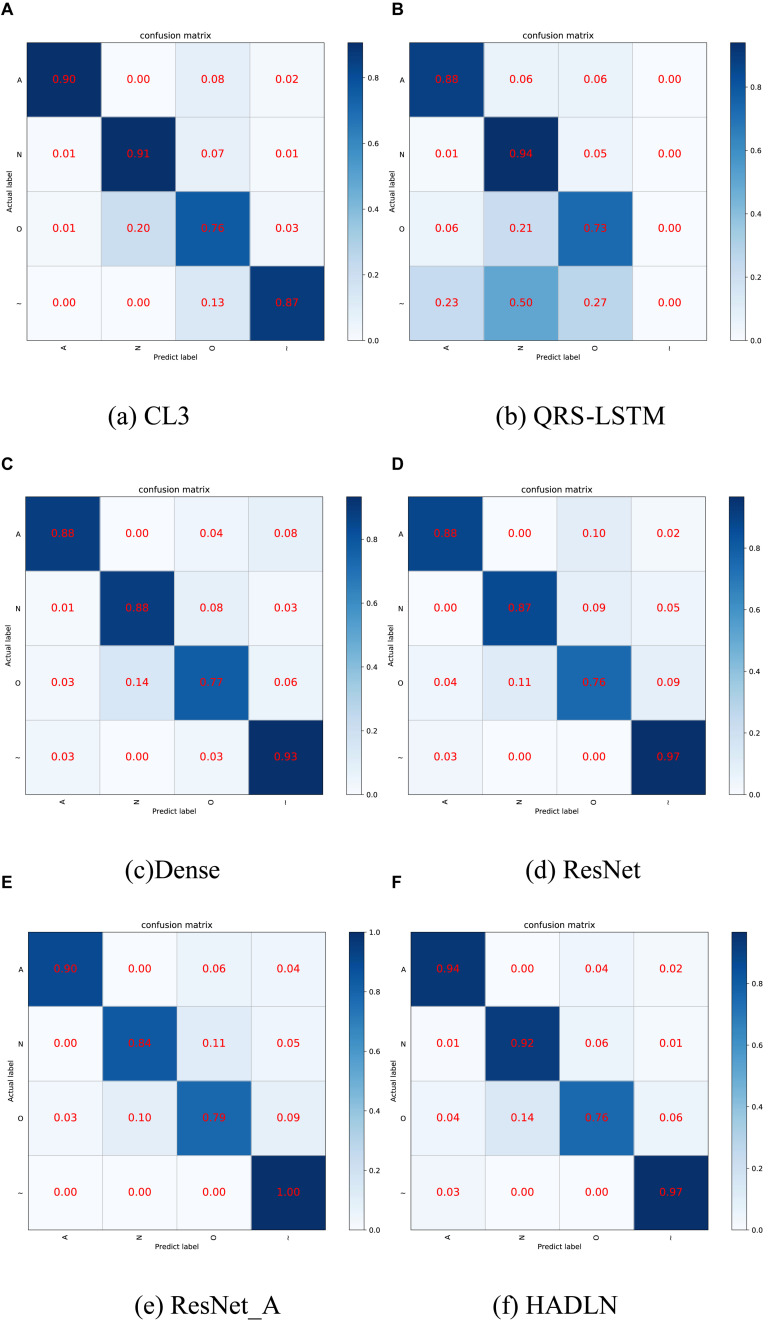
Confusion matrices by using different classification methods. **(A)** CL3 method, **(B)** QRS-LSTM method, **(C)** Dense method, **(D)** ResNet method, **(E)** ResNet_A method, and **(F)** HADLN method. The percentage of all records in each category is displayed on a color gradient scale.

As shown in Figure 6, the confusion matrices were used to illustrate the discordance between the predicted labels and the real labels by using different DNNs models. The results show that compared with the baseline model ResNet, the classification effect of normal (N) and atrial fibrillation (A) in HADLN is significantly improved by 5% and 6%. The classification effect of HADLN in atrial fibrillation (A) is generally higher than that of other contrast models.

## Discussion

Due to the limited size, each convolution operation can only cover a small neighborhood around the sequence, so that it cannot be easily captured the global features. Although after multilayer convolution stacking, compared with the single-layer CNN, more comprehensive features can be obtained. However, it still cannot make full use of the context information, resulting in a degradation in generalization ability. The advantage of the Bi-LSTM architecture is that it can learn long-term dependencies between sequences. Therefore, the Bi-LSTM network can be used to select the global feature from the original ECG signal. As shown in Table 4, the performance of HADLN is much higher than that of the model using only LSTM to classify QRS data, higher than the model of using only deep residual network. The above experimental results prove that the proposed HADLN method can adaptively discover hidden structures of different ECG signals and automatically learn relevant information, improving the accuracy of ECG data classification.

In this paper, attention mechanism is proposed to enhance the important information in the local feature information through different weightings and to weaken the interference information that may affect the classification performance. Therefore, the proposed HADLN method can improve the generalization ability, so as to extract comprehensive information and improve the classification accuracy obviously. The HADLN model proposed in this paper can adaptively discover hidden structures of different ECG signals and automatically learn relevant information, thereby improving the accuracy of ECG data classification. Through the attention mechanism, this deep learning model has better interpretability.

**FIGURE 7:**
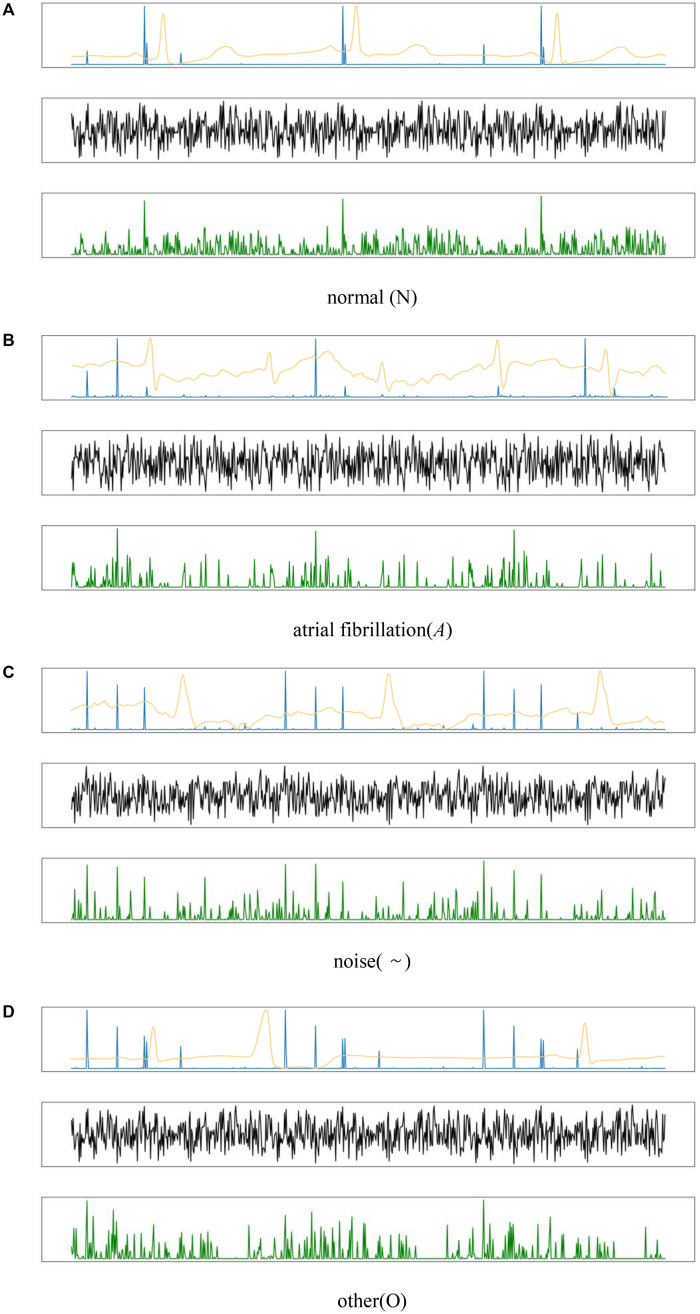
The output of feature mapping by using the different types for four kinds ECG signals: **(A)** normal, **(B)** atrial fibrillation, **(C)** noise, and **(D)** other. The yellow line is the ECG signal, the green line is mapping of the ResNet model, the black line is the mapping of ResNet_A model, and the blue line is the mapping of HADLN model.

As shown in the output mapping of the HADLN model represented by the blue line in Figure 7 (the weight of HADLN’s attention mechanism is similar to the output mapping), the normal category ECG signal reaches peak in the PR interval, and there is consistency between adjacent beats. The characteristic components of the ECG signal of atrial fibrillation category are concentrated on the abnormal P wave, and the RR interval is irregular. The ECG signal features of other category and noise category peaks are concentrated in multiple locations, which is far from the feature performance of normal category, and in the noise category, there are many dense and small peaks. Due to the normalization of the data, it is not very obvious in the visual display. At the same time, since some of the bands in the other category are approximately the same as the normal category, this is why the other category in the confusion matrix in Figure 7 have poor discriminating performance.

The black line in Figure 7 represents the output mapping of ResNet_A model whose weight is obtained from the ResNet output and weighted by itself. It can be found that the waveforms of various ECG signals are more complicated and fuzzier than the output mapping of ResNet, and the peaks are not prominent. This is very unfavorable for the final classification of the model. As shown in the experimental results of the above table, the accuracy of the ResNet_A model is far lower than that of ResNet and HADLN.

At the same time, by comparing the output mapping of ResNet represented by the green line in Figure 7 and the output mapping of attention mechanism of HADLN represented by the blue line, it can be found that the model proposed in this paper is finally achieved with different weights by adding the attention mechanism module. Enhancing important information in local feature information weakens the purpose of interference information that may affect classification performance. At the same time, through the attention mechanism, this deep learning model has a better explanation. It can be seen from the correct output mapping of the attention mechanism that the features extracted by this model are consistent with clinical judgments, indicating that HADLN has potential effectiveness in the recognition of most atrial fibrillation.

In recent years, many researchers were studying the problem of automatic ECG arrhythmia classification. He et al. (2019) proposed a new method for automatic classification of arrhythmias based on deep residual convolutional module and bidirectional LSTM module. Chu et al. (2019) used multilead CNN, LSTM network, and hand-crafted method to extract features. Yildirim et al. (2019) used convolutional auto-encoder LSTM to obtain 99.23%. Yao et al. (2020) combined CNN and LSTM to detect arrhythmia using varying lengths of ECG signals. Oh et al. (2018) combined CNN and LSTM to detect arrhythmia using varying lengths of ECG signals. The proposed HADLN method in this paper can classify ECGs signals with good performance. Although the optimized model provides an effective method for the automatic classification of ECG signals, it has not been tested by actual clinical diagnosis and application of actual patients. In addition, the model proposed in this paper are limited to the four major categories of cardiovascular disease, namely, atrial fibrillation (A), noise (∼), normal (N), and other (O), which make the model’s generalization in other fields have certain limitations.

## Conclusion

This paper proposed an HADLN method to classify four rhythm categories: normal (N), atrial fibrillation (A), other (O), and noise (∼). The proposed HADLN method makes full use of the advantages of ResNet and Bi-LSTM architecture to obtain fusion features containing local and global information and improve the interpretability of the model through the attention mechanism. Compared with the most advanced classification methods, it has great advantages. This method provides a promising way to improve the accuracy and interpretability of clinical applications. In future works, the proposed HADLN method will be used for arrhythmia classification to assist in clinical diagnosis.

## Data Availability Statement

The original contributions presented in the study are included in the article/supplementary material, further inquiries can be directed to the corresponding author/s.

## Author Contributions

JG and MJ: conceptualization, formal analysis, and methodology. MJ: resources, supervision, and project administration. JG, YL, and BW: software and visualization. JG: writing—original draft preparation. MJ and YL: writing—review and editing. LX and ZW: revising and correcting. JZ, ZW, and JG: clinical interpretation and discussion of findings and their relevance. All authors contributed to the article and approved the submitted version.

## Conflict of Interest

The authors declare that the research was conducted in the absence of any commercial or financial relationships that could be construed as a potential conflict of interest.
